# Virus–Bacteria Interactions: Implications and Potential for the Applied and Agricultural Sciences

**DOI:** 10.3390/v10020061

**Published:** 2018-02-02

**Authors:** Matthew D. Moore, Lee-Ann Jaykus

**Affiliations:** 1 Department of Food Science, University of Massachusetts, Amherst, MA 01003, USA; 2 Department of Food, Bioprocessing, and Nutrition Sciences, North Carolina State University, Raleigh, NC 27695, USA; lajaykus@ncsu.edu

**Keywords:** virus–bacteria interaction, agricultural sciences, translational medicine, foodborne pathogens, agronomy, influenza, norovirus

## Abstract

Eukaryotic virus–bacteria interactions have recently become an emerging topic of study due to multiple significant examples related to human pathogens of clinical interest. However, such omnipresent and likely important interactions for viruses and bacteria relevant to the applied and agricultural sciences have not been reviewed or compiled. The fundamental basis of this review is that these interactions have importance and deserve more investigation, as numerous potential consequences and applications arising from their discovery are relevant to the applied sciences. The purpose of this review is to highlight and summarize eukaryotic virus–bacteria findings in the food/water, horticultural, and animal sciences. In many cases in the agricultural sciences, mechanistic understandings of the effects of virus–bacteria interactions remain unstudied, and many studies solely focus on co-infections of bacterial and viral pathogens. Given recent findings relative to human viral pathogens, further research related to virus–bacteria interactions would likely result in numerous discoveries and beneficial applications.

## 1. Introduction

Bacteria and eukaryotic viruses have long been known to co-exist; however, identification of their relationships related to promoting or inhibiting each other’s presence in their eukaryotic hosts has only relatively recently garnered attention [1,2]. Numerous observations of co-infection among different pathogens—including transkingdom pathogens—have been well documented [3]; however, there are still many questions regarding the mechanisms and extent of how these pathogens and their interactions with other microbes result in differing levels of infection. A number of recent reviews on eukaryotic virus–bacteria interactions have suggested that these interactions are likely pervasive and have serious consequences for microbial pathogenesis and thus warrant further investigation. However, a lot of these reviews have basic microbiological focus on pathogens, their replication mechanisms, and interactions with the host. These important interactions have not been reviewed for microorganisms relevant to the applied and agricultural sciences. The basis of this review is that virus–bacteria interactions warrant more consideration and research in the applied and agricultural sciences, and may offer untapped potential for utilization in these fields. This review will highlight examples of these interactions in the agricultural sciences, their implications, and potential for utilization in the food/water, horticultural, and animal sciences.

## 2. Implications and Applications for Foodborne and Waterborne Pathogens

Food and waterborne pathogens impose a serious public health burden, and a number of food and waterborne viruses have been found to interact with host native enteric bacteria (Table 1). Likewise, these interactions have the potential to be utilized to enhance control of a number of enteric pathogens. Human noroviruses are a leading cause of acute viral gastroenteritis globally, and are estimated to cause about 685 million illnesses and over 210 thousand deaths annually [4]. One of the major hurdles in the study of human noroviruses had been the historic lack of an animal tissue culture model or ideal animal model for the viruses [5]. In 2014, Jones et al. [6] reported the first animal tissue culture model for productive infection of human noroviruses. In this case, Jones et al. reported the requirement of the gut bacteria *Enterobacter cloacae* or a synthesized norovirus putative carbohydrate receptor for infection of animal tissue, in this case human B cells. It had previously been reported that *E. cloacae* expresses carbohydrates very similar or equivalent to the human forms of carbohydrates traditionally suspected of being human norovirus receptors/co-receptors. In addition, *E. cloacae* was reported to bind human noroviruses in a specific manner, with the binding interaction confirmed by ELISA, electron microscopy, loss of binding of a mutant virus, and loss of binding with enzymatic cleavage [7]. Jones et al. [6] further expanded upon this finding by not only observing that *E. cloacae* and other enteric bacteria present in unfiltered human norovirus-containing stool were necessary for human norovirus replication in B cells, but also presented some evidence suggesting potential mechanisms behind the interaction. Specifically, using unfiltered stool significantly increased norovirus attachment to cells as compared to filtered stool. When synthetic receptor carbohydrates were added to filtered stool attachment was restored comparable to unfiltered, enteric bacteria-containing stool. Furthermore, evidence from co-cultures consisting of a top layer of intestinal epithelial cells with B cells below suggested that enteric bacteria may assist viral translocation across epithelial cells and into B cells, as only considerable increases in virus were seen in the B cell layer if unfiltered stool was used [6]. Further investigation into the binding of noroviruses to other enteric bacteria has been reported that may have potential applications in food and water research. Specifically, Rubio-del-Campo et al. [8] characterized the ability of a portion of two norovirus strains’ capsid proteins to bind different lactic acid bacteria, some of which were probiotic. Specifically, they observed binding for both human norovirus strains to probiotic and non-probiotic bacteria, as well as one Gram-negative bacterium (*Escherichia coli* Nissle 1917). Interestingly, Rubio-del-Campo et al. [8] observed that prior incubation with bacteria reduced virus capsid protein binding to cultured intestinal epithelial HT-29 cells but found that either adding bacteria to the cells prior to or in concert with introduction of capsid protein increased capsid protein binding [8], supporting Jones et al. [6]’s observation that resident enteric bacteria may increase viral attachment to host cells. Further investigation into the relationship between viruses and non-pathogenic bacteria or their cell membrane components may offer potential for therapeutic applications, as addition of other virus-binding components (e.g., lysed cells of bacteria “generally recognized as safe”) could be used as a therapeutic to reduce virus binding before or during symptom presentation, perhaps resulting in less severe symptoms or disease prevention.

In another study characterizing the scope of enteric bacteria-norovirus binding, Almand et al. [9] examined and quantified the degree of binding of three different strains of infectious human norovirus from stool to five representative bacteria isolated from human stool and two common lab strains. As with Rubio-del-Campo et al. [8], the binding of viruses seemed to occur at fairly high percentages for nearly all of the bacteria studied for all three human norovirus strains when using a suspension assay. Interestingly, one closely related human norovirus surrogate in the same family as norovirus (Tulane virus) selectively bound some bacteria while a plant virus related only in size and capsid properties (Turnip Crinkle Virus) bound none of the bacteria at appreciable levels [9]. This further supported the idea that noroviruses are binding putative receptor-like carbohydrates in bacteria, as Tulane virus selectively binds only certain types of the putative human norovirus carbohydrate receptors [10] as well as sialic acid [11]. In addition, the media used to culture bacteria significantly affected norovirus binding, with richer media reducing virus binding, in many cases by nearly 99% [9]. Further investigation into the mechanisms and behavior of enteric bacteria-norovirus binding, and the degree of binding that occurs in different conditions in the intestine throughout digestive process should be conducted given this finding. For five of the investigated bacteria, Almand et al. [9] report over 50% of viruses in solution were bound by bacteria, with some capture efficiencies at nearly 90% in optimal (minimal) media. This finding may offer promise in aiding more efficient detection of enteric virus contamination in food and environmental samples, as a small number of viruses must be concentrated from large, complex samples. Typically, nonspecific concentration methods also concentrate inhibitors, thus specific methods utilizing paramagnetic bead-based separation and washing (i.e., immunomagnetic separation) are also employed. However, these methods often offer very poor capture efficiency (<30% capture). Given the results by Almand et al. [9], application of norovirus-binding bacteria may offer a promising alternative for the concentration and detection or noroviruses from food and environmental samples.

Conversely some bacteria—including *E. cloacae*—have been reported to inhibit human norovirus infection in a gnotobiotic pig model [12,13]. In two reports, gnotobiotic pigs were colonized with either *E. cloacae*, *Lactobacillus rhamnosus* GG, or *Escherichia coli* Nissle 1917 and then infected with human norovirus. In one case, both *L. rhamnosus* and *E. coli* were mixed in a cocktail formulation and experimentally fed to the animals. In both sets of experiments, human norovirus shedding was markedly reduced and better maintenance of intestinal morphology was observed when the animals were pre-colonized with the bacteria [12,13]. Other experimental observations included immune activation of interferon gamma (IFN-γ) by bacteria and increased levels of intestinal IgA and IgG in bacteria-fed pigs compared to controls [13]. Unlike what was observed in vitro above, no infection of the B cells was observed although intestinal enterocytes appeared to be infected, consistent with another human norovirus tissue culture report [14]. It is possible that these observations are an artifact of the specific experimental system being used, as Jones et al. [6] utilize a mouse model in vivo with murine norovirus; and human B cells and murine macrophages for human and murine norovirus, respectively. One potential reason could be differences between immune response to viral infection between the two models. Generally, Type III IFN (IFN-Δ) has been implicated as the crucial factor in inhibiting murine noroviral persistence in epithelial cells [15,16]; although IFN-γ (Type II) has been implicated in control of murine norovirus infection through disrupting translation in a PKR-dependent manner [17], as Type I and Type II interferons are generally considered to inhibit systemic replication of murine norovirus. In the study with gnotobiotic pigs, Lei et al. [13] only followed IFN-γ levels as a broader characterization of IFN response would have been beyond the scope of the study. Lei et al. [13] also did not utilize *Enterobacter cloacae,* which was shown to generally inhibit norovirus infection in another study that did not follow IFN response [12]. Future work into the differences in IFN response of gnotobiotic pigs and mice to colonization with different bacteria (including *E. cloacae*) in the context of norovirus infection would likely be valuable. Undoubtedly, more work is needed to better understand human norovirus pathogenesis and the role(s) different enteric bacteria may play.

Similar discrepancies as norovirus have also been observed with rotavirus infection. Some studies have suggested that colonization with certain bacteria has a protective effect on rotavirus infection. In a placebo-controlled, double blind study, Saavedra et al. [18] found that infants admitted to a chronic care hospital who were given formula supplemented with *Bifidobacterium bifidum* and *Streptococcus thermophilus* were significantly less likely to shed rotavirus during their admission than infants given formula alone. It should be noted that the sample size of the study was fairly small, however. Subsequent study in gnotobiotic pigs has also suggested a potential protective effect against rotavirus infection in a bacterial species-dependent manner. Specifically, colonization of pigs with *E. coli* Nissle 1917 appeared to reduce rotavirus shedding and confer greater protection by increasing plasmacytoid dendritic cells and natural killer (NK)-cell activity in vivo as well as interleukin (IL)-12, IFN-α, and IL-10 in vitro, while *Lactobacillus rhamnosus* GG had no effect [19,20]. Alternatively, a study with mouse rotavirus suggested bacterial colonization may enhance rotavirus infection. In this case, Uchiyama et al. [21] found that depletion of the microbiota using antibiotics in mice reduced rotavirus infectivity by over 40% and delayed the onset of infection. Interestingly, the ratios of positive and negative stranded RNA were not affected by microbial depletion, suggesting that the reduction in infectivity was due to the binding and uncoating stages of infection [21]. Treatment with antibiotics also resulted in a more robust rotavirus IgA antibody response [21], which is the opposite of the enhancement of IgA response observed with *E. coli* Nissle 1917 in gnotobiotic pigs [20]. It should be noted that Uchiyama et al. [21] was testing general microbiota; given the observation that different bacterial species may have different specific effects on rotavirus infection, the discrepancies observed could be due in part to the composition of the mouse microbiota that was removed. Alternatively, the discrepancy could be due to differences in the mouse rotavirus/mouse and human rotavirus/gnotobiotic pig models used. Certainly, future work should focus on the differential effects of certain bacteria on rotavirus infection.

In addition to the studies discussed above on bacterial protection against norovirus and rotavirus infection, multiple other examples of probiotics demonstrating antiviral activity through different mechanisms are reviewed by Al Kassa et al. [22]. For the purposes of this review, two additional examples of antiviral probiotic interactions with different potential mechanisms are discussed. In addition to immune activation as in the cases above, probiotic bacteria can display antiviral activity by directly binding/capturing virus and/or competing for cell adhesion. This was the case in a study utilizing porcine intestinal epithelial cells with vesicular stomatitis virus as a model virus, as a panel of multiple probiotic bacteria was capable of reducing virus infectivity up to 60% when bacteria were pre-incubated with host cells. Additionally, the bacteria were found to be excreting antiviral compounds, as bacterial supernatant was capable of reducing viral titer considerably in vitro [23]. Multiple potential components of the bacterial supernatant could be responsible for the antiviral activity observed. One group of proteinaceous molecules produced by bacteria, known as bacteriocins, display antiviral activity. Usually active against related bacteria, Enterocin CRL35, a bacteriocin produced by *Enterococcus faecium* CRL35, was demonstrated to reduce replication of herpes simplex virus 1 and 2 by about 1 log_10_ in vitro. Evidence suggested that the bateriocin inhibited synthesis of a viral glycoprotein necessary for infection and replication [24]. The ability of enteric bacteria to bind virus, prevent its adherence to host cells, and/or inhibit some aspect of the viral infection process may all have potential therapeutic value.

Another group of food and waterborne virus–bacteria interactions has implications for control of these viruses and offers new considerations in investigating treatments. As mentioned above, enteric bacteria have been reported to assist infection in numerous ways by multiple enteric viruses [1,2]; including an initial report suggesting that numerous enteric bacteria increase poliovirus co-infection efficiency and increase fitness by promoting recombination [25]. Evidence also suggests that binding to enteric bacteria may increase the heat tolerance of certain enteric viruses. Initial evidence of this was first reported by Kuss et al. [26], who observed increased replication of poliovirus treated with *N-*acteylglucosamine-containing bacterial surface polysaccharides (e.g., lipopolysaccharide and peptidoglycan) at an elevated temperature (42 °C) compared to PBS-treated poliovirus. This work was further advanced by Robinson et al. [27], who found that bacterial *N-*acteylglucosamine-containing polysaccharides enhanced poliovirus stability to bleach treatment. Additionally, the temperature at which poliovirus undergoes conformational change and releases RNA was found to be elevated in a dose-dependent manner when exposed to bacterial lipopolysaccharide. Interestingly, a mutant poliovirus strain with reduced bacteria binding ability was isolated, and found to be less environmentally stable than wild type poliovirus [27]. Similar results were observed for human norovirus by Li et al. [28], who showed that exposure of different human norovirus capsids to two norovirus-binding *E. coli* strains increased the receptor-binding ability of the capsids when treated with heat (90 °C, 2 min) compared to a strain of *E. coli* that did not bind human norovirus. Because cooking and bleach treatment are common control points for pathogens in food systems, the observation that some bacteria—likely also present along with virus in fecally-contaminated foods—may increase enteric virus stability is worth serious consideration. This is because nearly all studies on the efficacy of different heat and chemical treatments are performed with only virus or artificial “soil” loads that do not contain bacterial polysaccharides as a component. Future work that re-evaluates potential enhancement of enteric virus resistance to some of these treatments may be worth consideration.

## 3. Applications and Promise in the Horticultural Sciences

Although a lot of mechanistic research related to eukaryotic-virus bacteria interactions has been performed in the food/water field, less work has been performed related to the horticultural sciences—specifically regarding plant virus–bacteria interactions. A large amount of literature exists studying the effects of both the abiotic (i.e., drought, heat, etc.) and biotic (pests, fungal and microbial pathogens, etc.) stresses that affect plants—including interactions and the effects within and between these different stresses—but little work has been performed investigating bacteria–virus interactions within this field (see review by Lamichhane and Venturi [29]). However, other dual pathogen interactions involving plant viruses have been studied and reported, such as virus–virus interactions [30].

A study by Tollenaere et al. [31] chose to survey and study rice (*Oryza* spp.) in Africa because it is being rapidly planted to meet a growing demand. The rationale for this work was that evidence exists suggesting that plants may simultaneously host multiple pathogens, and this could affect the dynamics of host (plant) resistance and pathogen evolution in a possibly different way than studying one plant–one pathogen interaction (Table 2). In the work, Tollenaere et al. [31] survey 30 different rice fields across three different irrigation zones in Africa (Burkina Faso) for two common pathogens: rice yellow mottle virus and *Xanthomonas oryzae*. They also analyzed the effects of the two pathogens on each other in co-infected plants (both qualitative and quantitative), and investigated potential mechanisms for any observed effects that the two pathogens may have on each other. Not surprisingly, both the virus and bacteria were found in a large number of plants, and nearly a fifth of all plants tested (18.8%) were positive for both virus and bacteria. When observing the phenotypes of the plants, generally bacterial pathogenic symptoms were visible and viral symptoms reduced. Experimental co-infection of plants was conducted using two different virus strains isolated from different areas. Quantitative PCR was used to determine the relative levels of the pathogens in co-infected plants, with bacterial load being significantly increased when co-infecting with a virus. The levels of one strain of virus were reduced in the presence of *X. oryzae* but not the other strain. Tollenaere et al. [31] present evidence suggesting that the mechanism of viral suppression by *X. oryzae* was linked to promotion of RNA silencing and targeting of viral genomes by the plants. This effect was observed by experimental co-infection of plants with virus, wild type bacteria, and two mutant versions of bacteria lacking either a Transcription Activator-like Effector (TALE) that interacts with the promoter of an important *Oryza* spp. protein in the anti-viral RNA silencing pathway or a Type III secretor used to inject TALEs into the plant. As expected, the wild type *X. oryzae* considerably reduced viral levels by over 60% in the plant relative to a virus-only infected plant, while significantly less reduction of viral levels was seen in the two bacterial mutant co-infected plants [31].

In a study by Shapiro et al. [32], connections between a plant virus, bacteria, and pests (cucumber beetles) were examined to explain the observation that virus-infected plants were less frequently infected by bacterial wilt than uninfected plants. Salicyclic Acid, a phytohormone that is generated as a defense to pathogens, was induced at high levels with infection by zucchini yellow mosaic virus, and even induced at higher levels with co-infection by virus and *Erwinia tracheiphila* but not *E. tracheiphila* alone. Infection of the plants by virus slightly delayed onset of bacterial wilt caused by *E. tracheiphila* by one day in inoculation experiments. However, additional experiments suggested that virus infection resulted in reduced visitation by cucumber beetles, which are vectors for *E. tracheiphila.* Thus, the mechanism by which zucchini yellow mosaic virus infection reduces the rates of bacterial wilt is likely indirect in part by reducing visitation by bacterial beetle vectors. The specific mechanism by which the virus reduces visitation by beetles was not reported but suspected to be induction of plant traits that make them less desirable to beetles, and likely future research into the mechanism may be of interest for the purposes of pesticide alternatives [32].

Another interaction in agronomy involves the use of a compound that has beneficial effects on plants, but may have peripheral consequences for animal viruses based on observations of animal virus–bacteria interactions. Specifically, chitin and modified chitin compounds have been reported to stimulate plant growth, assist nutrition, promote beneficial microorganisms, and control different plant pathogens and pests (reviewed in Sharp [43]). Chitin is a polymer that contains multiple *N-*acetylglucosamine units, and has been shown to interact and specifically increase the stability of polioviruses discussed above [26,27]. Use of chitin in crop production could theoretically result in potentially stabilizing enteric viruses, or result in runoff that could end up in irrigation water that is used to treat produce. However, the effect of chitin on poliovirus attachment and infectivity at normal temperatures, interaction with polioviruses, or the effect of chitin on other enteric viruses of concern was not investigated [27]. Considering that a notable amount of produce has been associated with foodborne enteric virus outbreaks [44,45,46], and that a lot of produce is eaten raw and not subjected to a cooking kill step, the form of chitin used and its effects on enteric viruses may warrant some consideration. For example, deacetylation of chitin results in chitosan, which has displayed antimicrobial activity including activity against plant viral pathogens and animal enteric viruses, with mixed but positive results on different virus surrogates [43,47,48,49]. Additionally, chitosan appeared not to have any beneficial effect stabilizing polioviruses to heat; however, chitosan’s other effects on other enteric viruses, viral attachment, and overall viral replication was not investigated [27]. Taking these findings into consideration, chitosan would likely be preferable for agricultural use; however, more study on the effects of chitin and chitosan and their effects on bacteria and viruses is needed, especially in understanding the mechanisms behind observed antibacterial and antiviral activity [43].

A number of other indirect interactions between plant viruses and bacteria have been investigated in the case of bacterial protection against viral pathogens. Specifically, a good deal of work has been reported on how different rhizobacteria protect against multiple plant pathogens, including viruses. Generally, the mechanism by which this occurs is through induction of systemic resistance in the plants (reviewed by [33,34]). This is generally the case for protection of multiple plants against different plant viruses. In one case, different combinations of different *Bacillus* species have been shown to have an antiviral effect on cucumber mosaic virus in both a model plant (*Arabidopsis thaliana*) [36,37] and produce (tomato) [35]. Some results suggested that protection against cucumber mosaic virus is acquired through a different pathway than one induced by salicyclic acid or downstream gene *NPR1* that elicit resistance against bacteria and fungi [36]. However, in another case, some evidence suggested that production of salicyclic acid by rhizobacteria may more directly induce systemic resistance of some plants. Specifically, biosynthetic genes from antiviral *Pseudomonas aeruginosa* were cloned into vectors and then transformed into a *Pseudomonas fluorescens* strain whose wild type did not produce salicyclic acid and was not efficient at inducing systemic resistance in plants. Cloning of the genes resulted in production of salicyclic acid by the bacteria, and induced systemic resistance against tobacco necrosis virus [38]. In another study investigating the influence of naturally occurring rhizobacteria on plant growth and resistance to disease, native bacteria isolated from the rhizosphere of the hot pepper plant were tested for their effects on plant growth and resistance to tobacco mosaic tobamovirus. Although strain-dependent, a number of bacterial isolates were found to increase growth and induce resistance to the virus, resulting in a number of favorable phenotypic traits such as increased height, flower and fruit number, and fruit flesh weight [39]. Although a lot of the reviewed work has been focused on bacteria isolated from the rhizosphere, leaf-associated bacteria have recently been demonstrated to exhibit a protective effect against viruses. In this case, a leaf-associated *Bacillus amyloliquefaciens* strain isolated from the leaf of a cherry tree was found to decrease the levels and severity of cucumber mosaic virus when sprayed onto pepper and tobacco plants. Evidence that the bacterial application induced systemic resistance in the plants was observed via upregulation of genes in pepper associated with plant defenses. Reduced levels of the naturally-circulating pepper mottle virus and broad bean wilt virus were observed in bacteria-treated peppers compared to controls, further supporting induction of systemic resistance [40].

In addition to having an effect on plant viruses, some evidence has been reported that native plant bacteria can affect animal viruses. In an interesting study by Esseili et al. [41], counts of the aerobic cultivable bacteria present on lettuce and spinach were compared with survival of human noroviruses along with some of its cultivable surrogates. Survival of both human noroviruses and its cultivable surrogates significantly positively correlated with bacterial counts in spinach. This was not the case for human norovirus survival and bacterial counts in lettuce [41]. However, characterization of the specific bacterial communities in the two plants was not investigated, as it was beyond the scope of the study. These findings suggest that future work to characterize potential native bacterial species/genera on plants that may assist viral survival would be valuable. Another study focused on the effects of bacteria present in manure on inactivating hepatitis A virus. Specifically, 31 strains of bacteria isolated from manure were evaluated for their ability to reduce the titer of hepatitis A at 37 °C over the course of multiple days. Ten isolated strains were capable of reducing hepatitis A titers by 1 log_10_ in less than 10 days. The mechanism of the inactivation was not determined, though evidence suggested that it was not due to an increase in pH or enzymatic action [42]. Although an intriguing potential means of biological control of foodborne pathogens in sewage and fertilizer treatment, further work identifying bacteria that are more immediately effective and lethal or additional treatment to further reduce viral titer would need to be identified.

There are still many additional potential areas of future investigation that may be valuable to the horticultural community. A recent body of work related to the potential positive roles of some eukaryotic viruses as symbionts in their hosts has been convincingly argued for both animal and plant viruses [50,51]. One example involves conferring heat resistance in tropical panic grass that allows for the grass to grow in extreme conditions. Specifically, the fungal endophyte *Curvularia protuberate* is required for the *Dichanthelium lanuginosum* grass to grow in Yellowstone National Park. However, a novel dsRNA virus was discovered in *C. protuberate*, and removal of the virus from the grass resulted in loss of heat tolerance, while reintroduction of the virus with a marker rescued heat resistance of the grass [52]. On the other hand, there are a number of endogeneous retroviruses in plants that occasionally exogenize in response to stress and cause infection. For example, banana streak virus is a pararetrovirus present in the genomes of banana (*Musa* spp.), and multiple instances of the virus capable of doing this have been reported [53,54]. The breadth and specific nature of the stresses that cause endogenous banana streak virus to exogenize is not well known; however, the role of potential bacterial stress may be worth investigating given the antiviral stimulation caused by some native bacteria discussed above, as well as potential bacterial pathogenic infection [55]. Once exogenized, infectious viral particles are transmitted by mealybugs [56]; the potential role of native microbiota in assisting binding and dissemination of these viruses to other plants may be of value. One other final application of viruses in horticulture is the potential for identification of bacteria that assist viruses of pests. The possibility of utilizing viruses as a natural form of pest control has been investigated [57], but the potential role of bacteria assisting or preventing viral infections has not been widely investigated. In the model insect *Drosophila melanogaster*, *Wolbachia* spp. have been demonstrated to reduce the load and increase *D. melanogaster*’s resistance to *Drosophila* C virus, Flock House virus, and Nora virus [58]. The possibility of other bacteria that have the opposite effect as *Wolbachia* spp. also exists and may warrant investigation. Multiple different areas of investigation regarding bacteria–virus interactions in horticulture may offer better understanding of plants as holobionts and offer numerous avenues of potential beneficial application.

## 4. Virus–Bacteria Relationships in Food Animals

Much like the horticultural sciences, a number of papers noting interactions between bacteria and viruses of concern in aquaculture and livestock have been reported (Table 3). Generally, the mechanisms and nature of the interactions are less well known. Co-infection of different virus and bacterial pathogens seems to comprise a majority of the reports observing interactions/effects between bacteria and viruses. In general, the effect of native microflora on potential viruses is less well studied with the exception of the effect of lactic acid bacteria on potential pathogens.

Like was observed by Lei et al. [13] in gnotobiotic pigs discussed above, lactic acid bacteria in the guts of fish appear to also provide a potential protective effect against certain microbial pathogens, though not much work has been done on viral pathogens (reviewed in [69]). In one report, grouper fed different doses (0–10^10^ cfu/kg) *Lactobacillus plantarum* had survival rates increased as much has 26.7% when challenged with grouper iridovirus. Some evidence indicated that *L. plantarum* stimulated the immune system of the fish, which was one of the suspected mechanisms of protection. Interestingly, an increase in fish survival rate corresponded to higher *L. plantarum* doses from 0–10^8^ cfu/kg, but the survival rate goes back down at 10^10^ cfu/kg [59]. Another study found direct antiviral effects of dextrans isolated from the exopolysaccharide layers (EPS) of *Lactobacillus sakei* MN1 and *Leuconostoc mesenteroides* RTF10 on two salmonid fish viruses, infectious pancreatic necrosis virus (IPNV) and infectious hematopoietic necrosis virus (IHNV). Commercial, purified dextrans were also tested, with only the largest dextran (T2000) displaying antiviral activity. An antiviral effect for EPS was observed in vitro via 50% tissue culture infective dose (TCID_50_) by addition of EPS after viral introduction to cells, as well as in vivo in rainbow trout with *L. sakei* EPS*.* Although the specific mechanism was not conclusively determined, evidence suggested that introduction of the EPS activated an antiviral immune response, as first levels of poly I:C then IFN-1 spiked in treated fish relative to controls (innate response) followed by a spike in IFN-γ (adaptive response) [60].

A number of studies describing bacterial and viral pathogens interacting in co-infections in aquaculture have been reported, and are reviewed by Kotob et al. [61]. For the purposes of this review, some of these studies will be briefly summarized as examples. Unlike lactic acid bacteria, many reports of co-infection by a bacterial and viral pathogen suggest a synergistic effect. Aquabirnaviruses infect a number of fish, but generally do not normally cause symptoms. Pakingking et al. [62] experimentally tested the survival rates of flounder infected with or without aquabirnavirus and bacterial pathogens *Edwardsiella tarda* and/or *Streptococcus iniae*. Fish that were co-infected with both the birnavirus and a bacteria had a 20–35% higher mortality rate than those infected with a bacterium alone [62]. A similar result is presented by Oh et al. [70], as aquabirnavirus was found to increase mortality of *Vibrio harveyi* or *E. tarda* in Olive flounder when intraperitoneally injected. A survey of birnaviruses in Olive flounder in Korean waters by Jung et al. [71] presents some evidence that co-infection of aquabirnaviruses and bacterial pathogens does occur in nature. As was observed with aquabirnaviruses, infectious pancreatic necrosis virus infection in concert with *Vibrio salmonicida* [63] and *Vibrio carchariae* [64] resulted in high mortality with Atlantic salmon and grouper, respectively. Intriguingly, prior infection with the virus resulted in the opposite effect with regards to another viral pathogen, infectious salmon anemia virus, as infection with infectious pancreatic necrosis virus prior to infection with infectious salmon anemia virus resulted in lower mortality than infectious salmon anemia virus alone. The mechanism for this observation was not investigated. In addition to fish, shrimp have also been victim to enhanced pathogenesis upon bacteria-virus co-infection. Specifically, infection of shrimp with white spot syndrome virus prior to *Vibrio campbellii* resulted in accelerated mortality and significantly higher levels of *V. campbellii* in virus-infected shrimp compared to uninfected shrimp [65].

Virus–bacteria interactions in livestock have also been reported, with similar observations of synergy between bacterial and viral pathogens. One of the more historically documented instances of this is Bovine Respiratory Disease Complex, sometimes called “shipping fever”. This disease is multifactorial, involving multiple potential etiological agents, both viral and bacterial. One specific combination of these that has been studied is the relationship between bovine herpesvirus-1 (BHV-1) and *Mannheimia haemolytica* (formerly *Pasteurella haemolytica*) [67,72]. Generally, viral infection results in immunosuppression that can allow for serious secondary infection that causes pneumonia and high mortality. *M. haemolytica* produces a leukotoxin that binds the host leukocytes and activates cytolysis of the cells. Infection with BHV-1 in cattle caused increased expression of the host receptors the leukotoxin targets, and thus resulted in increased leukotoxin binding and leukocyte death. Additionally, BHV-1 infection increased the number of leukocytes in the cattle [66]. Another virus implicated in Bovine Respiratory Disease Complex, bovine viral diarrhea virus, also was observed to result in immunosuppressed cattle and increased *M. haemoltyica* infection [67,68].

## 5. Conclusions

As can be seen, a number of studies have demonstrated the importance of virus–bacteria interactions in the agricultural and applied sciences. However, many of the studies either lack specific mechanistic insight that may prove valuable for multiple applications in the fields discussed. In these fields, a lot of work has been directed on interactions between viruses and bacteria in co-infected hosts; however, less work has been performed on the role of the host and environmental microbiota. One exception has been the beneficial impact of lactic acid bacteria and rhizobacteria in reducing the severity of viral disease, generally through indirect routes like immune activation. Given the numerous recent findings presented of more direct interactions between viruses and bacteria in humans, future exploratory work of additional direct virus-bacteria interactions in the applied sciences may prove valuable for functional application. As the rapidly growing field of eukaryotic virus–bacteria interactions evolves, multiple potential discoveries are likely to be made that will be valuable for both understanding fundamental processes and have functional applications in the agricultural sciences.

## Figures and Tables

**Table 1:** Selected reports on eukaryotic virus–bacteria interactions in food and water sciences.

Virus(es)	Bacteria	Interactions	Reference
Human norovirus, murine norovirus	*Enterobacter cloacae;* Unidentified bacteria in unfiltered stool	Norovirus infection of B cells assisted by bacteria; Viral attachment to host cells increased by presence of bacteria; bacteria may assist viral translocation across epithelial cells	[6]
Human norovirus	*Enterobacter cloacae*	Human noroviruses bind bacteria; bacteria expresses similar carbohydrates to human versions historically suspected of being receptors	[7]
Human norovirus	*E. cloacae*, *Escherichia coli* Nissle 1917, *Lactobacillus rhamnosus* GG	Reduced viral shedding was observed in gnotobiotic pigs colonized with bacteria; potentially reduced viral infection via innate and adaptive immune activation	[12,13]
Human norovirus capsid subdomains	10 lactic acid bacteria (probiotic and non-probiotic), *E. coli* Nissle 1917	Observe some degree of binding of virus proteins to all 11 bacteria; viral binding to intestinal cell line (HT-29) increased or decreased with introducton of bacteria depending on whether bacteria are pre-incubated with virus before introduction to cells	[8]
Human norovirus; Tulane virus	5 representative enteric bacterial isolates from stool, *E. cloacae*, *Staphylococcus aureus*	Observe and quantify binding of different infectious human norovirus strains to 7 enteric bacteria, showing binding to most strains at high efficiency; find binding is considerably affected by bacterial culture media; only selective binding to certain bacteria for related norovirus surrogate Tulane virus	[9]
Poliovirus	41 bacterial strains scanned	Poliovirus bound most bacterial strains; viral attachment to host cells enhanced by bacteria; some evidence bacterial co-infection increased viral co-infection efficiency and promoted viral recombination	[25]
Poliovirus	*N-*acetylglucosamine containing bacterial polysaccharides (lipopolysaccharide and peptidoglycan)	Exposure of virus to lipopolysaccharide and peptidoglycan increased virion stability/replication at elevated temperature (42 °C) and after exposure to bleach; evidence that exposure to these polysaccharides affects capsid conformational change and RNA release	[26,27]
Human norovirus	2 *E. coli* strains that bind virus, 1 *E. coli* strain with reduced binding	Found some evidence suggesting that exposure of virus capsids to virus-binding strains increased stability of capsid after heat treatment (90 °C, 2 min) compared to reduced binding *E. coli*	[28]
Multiple viruses	Multiple lactic acid bacteria	A review of antiviral effects of lactic acid bacteria through multiple mechanisms, including immune activation, bacteriocin inactivation of virus, and direct bacterial binding/capture by bacteria	[22]
Rotavirus	*E. coli* Nissle 1917, *L. rhamnosus* GG	Colonization with *E. coli* Nissle 1917 lowered rotavirus infectivity and enhanced innate and humoral immune response in gnotobiotic pigs	[19,20]
Rotavirus	*Bifidobacterium bifidum*, *Streptococus thermophilus*	Infants fed formula supplemented with probiotic cocktail displayed significantly less frequent diarrheal episodes and rotavirus shedding	[18]
Rotavirus	Unidentified bacterial microbiota	Microbiota depletion in mice by administration of antibiotics reduced rotavirus infectivity; likely due to less enhancement of viral binding and uncoating stage; microbiota-depleted mice diplayed more robust IgA response to rotavirus than control	[21]

**Table 2:** Selected reports of eukaryotic virus–bacteria interactions in the horticultural sciences.

Virus(es)	Bacteria	Interactions	Reference
Rice yellow mottle virus	*Xanthomonas oryzae*	Survey of numerous rice fields in Africa found 18.8% of sampled plants had indications of co-infection; Presence of bacterial pathogen in co-infection significantly reduced the viral titers in the rice; evidence that *X. oryzae* promotes antiviral RNA silencing pathway	[31]
Zucchini yellow mosaic virus	*Erwinia tracheiphila*	The wilt caused by *E. tracheiphila* generally reduced in virus-infected plants; some evidence viral infection induces phytohormone (salicyclic acid) in plants that causes phenotypic changes in plant that reduces plant attractiveness to cucumber beetle vectors that carry *E. tracheiphila*	[32]
Different plant viruses	Different rhizobacteria	Two reviews covering how rhizobacteria promote resistance to different plant pathogens	[33,34]
Cucumber mosaic virus	Combinations of *Bacillus* spp.	Application of bacteria has antiviral effect in *Arabidopsis thaliana* and tomato; mechanism is likely independent of salicyclic acid in one study	[35,36,37]
Tobacco necrosis virus	*Pseudomonas aeruginosa, Pseudomonas fluorescens*	Some evidence that *P. aeruginosa* may directly produce salicyclic acid to aid plant resistance to virus; when salicyclic acid-producing enzymes cloned into *P. fluorescens* strain that did not produce it or have antiviral activity, *P. fluorscens* demonstrated some viral inhibition	[38]
Tobacco mosaic tobamovirus	Multiple rhizobacteria isolated from hot pepper	Some isolated strains showed antiviral effect and resulted in plants with favorable traits: increased height, flower and fruit number, and fruit flesh weight	[39]
Cucumber mosaic virus	*Bacillus amyloliquefaciens*	*B. amyloliquefaciens* isolated from cherry tree leaf decreased severity and levels of virus when sprayed onto pepper and tobacco plants; also reduced naturally circulating pepper mottle virus and broad bean wilt virus in peppers	[40]
Human norovirus	Cultivable aerobic bacteria present in lettuce and spinach	Survival of human noroviruses in spinach significantly positively corresponded to bacterial levels; however not the case for lettuce	[41]
Hepatitis A	31 strains of bacteria isolated from manure	10 of the isolated strains reduced virus titers by >1 log_10_ in less than 10 days at 37 °C	[42]

**Table 3:** Eukaryotic virus–bacteria interactions in food animals.

Virus(es)	Bacteria	Interactions	Reference
Grouper iridovirus	*Lactobacillus plantarum*	Found that grouper fed different doses of bacteria had a generally higher survival rate to the virus	[59]
Infectious pancreatic necrosis virus (IPNV), infectious hematopoietic necrosis virus (IHNV)	Dextrans isolated from the exopolysaccharide (EPS) of *Lactobacillus sakei* MN1, *Leuconostoc mesenteroides* RTF10	Antiviral effect of the EPS and one commercial dextran (T2000) observed in vitro and in rainbow trout with *L. sakei* EPS; some evidence that mechanism was innate and adaptive immune activation	[60]
Multiple fish viruses	Multiple bacterial fish pathogens	A review of co-infections and their interactions in fish.	[61]
Aquabirnaviruses	*Edwardsiella tarda*, *Streptococcus inae*, *Vibrio harveyi*	Co-infection of a bacterial pathogen with virus resulted in increased mortality rates in different flounder	[62]
Infectious pancreatic necrosis virus	*Vibrio salmonicida*, *Vibrio carchariae*	Virus–bacterial co-infection resulted in higher mortality than infection by single pathogen in Atlantic salmon and grouper	[63,64]
White spot syndrome virus	*Vibrio campbellii*	Co-infection with virus and bacteria resulted in significantly higher mortality and levels of bacteria compared to shrimp without virus	[65]
Bovine herpesvirus 1, bovine viral diarrhea virus	*Manheimia haemolytica*	Viral infection results in immunosuppression that enables secondary infection; bovine herpesvirus infection of cattle resulted in increased leukocytes and receptors on leukocytes for an *M. haemolytica* leukotoxin to bind that results in leukocyte death	[66,67,68]
